# Gold nanoparticles synthesis using *Gymnosporia montana* L. and its biological profile: a pioneer report

**DOI:** 10.1186/s43141-023-00525-6

**Published:** 2023-06-26

**Authors:** Krishnakumari N. Patel, Pooja G. Trivedi, Milan S. Thakar, Kush V. Prajapati, Dhruv K. Prajapati, Gaurang M. Sindhav

**Affiliations:** Department of Zoology, BMT, HGC & WBC, University School of Sciences, Gujarat University, Ahmedabad, India

**Keywords:** Gold nanoparticles, *Gymnosporia montana* L., Spectroscopy, DNA, Cytotoxicity

## Abstract

**Background:**

The forming, blending, and characterization of materials at a size of one billionth of a meter or less is referred to as nanotechnology. The objective of the current study was to synthesize ecologically friendly gold nanoparticles (AuNPs) from *Gymnosporia montana* L. (*G. montana*) leaf extract, characterize them, assess their interaction with different types of deoxyribonucleic acid (DNA), and investigate their antioxidant and toxic capabilities.

**Results:**

The biosynthesized AuNPs presence was validated by a color change from yellow to reddish pink as well as using UV–visible spectrophotometer. Fourier transform infrared (FTIR) spectroscopy analysis showed the presence of phytoconstituents like, alcohols, phenols, and nitro compounds responsible for the reduction of AuNPs. Zeta sizer and zeta potential of 559.6 d. nm and − 4.5 mV, respectively, demonstrated potential stability. With an average size between 10 and 50 nm, X-ray diffraction (XRD), and high-resolution transmission electron microscope (HR-TEM), revealed the crystalline formation of AuNPs. Surface topology with 3D characterization, irregular spherical shape, and size with 6.48 nm of AuNPs was determined with the help of an atomic force microscope (AFM). AuNPs with some irregular and spherical shapes, and sizes between 2 and 20 nm, were revealed by field emission scanning electron microscope (FESEM) investigation. Shifts in the spectrum were visible when the bioavailability of AuNPs with calf-thymus DNA (CT-DNA) and Herring sperm DNA (HS-DNA) was tested. Additionally, the DNA nicking assay’s interaction with pBR322 DNA confirmed its physiochemical and antioxidant properties. The same was also found by using a 2,2-diphenyl-1-picrylhydrazyl (DPPH) assay, which showed a 70–80% inhibition rate. Finally, 3-(4,5-dimethylthiazol-2-yl)-2,5-diphenyl-2H-tetrazolium bromide (MTT) assay revealed that viability decreased with increasing dosage, going from 77.74 to 46.99% on MCF-7 cell line.

**Conclusion:**

Synthesizing AuNPs through biogenic processes and adopting *G. montana* for the first time revealed potential DNA interaction, antioxidant, and cytotoxicity capabilities. Thus, opening new possibilities in the turf of therapeutics as well as in other areas.

**Graphical Abstract:**

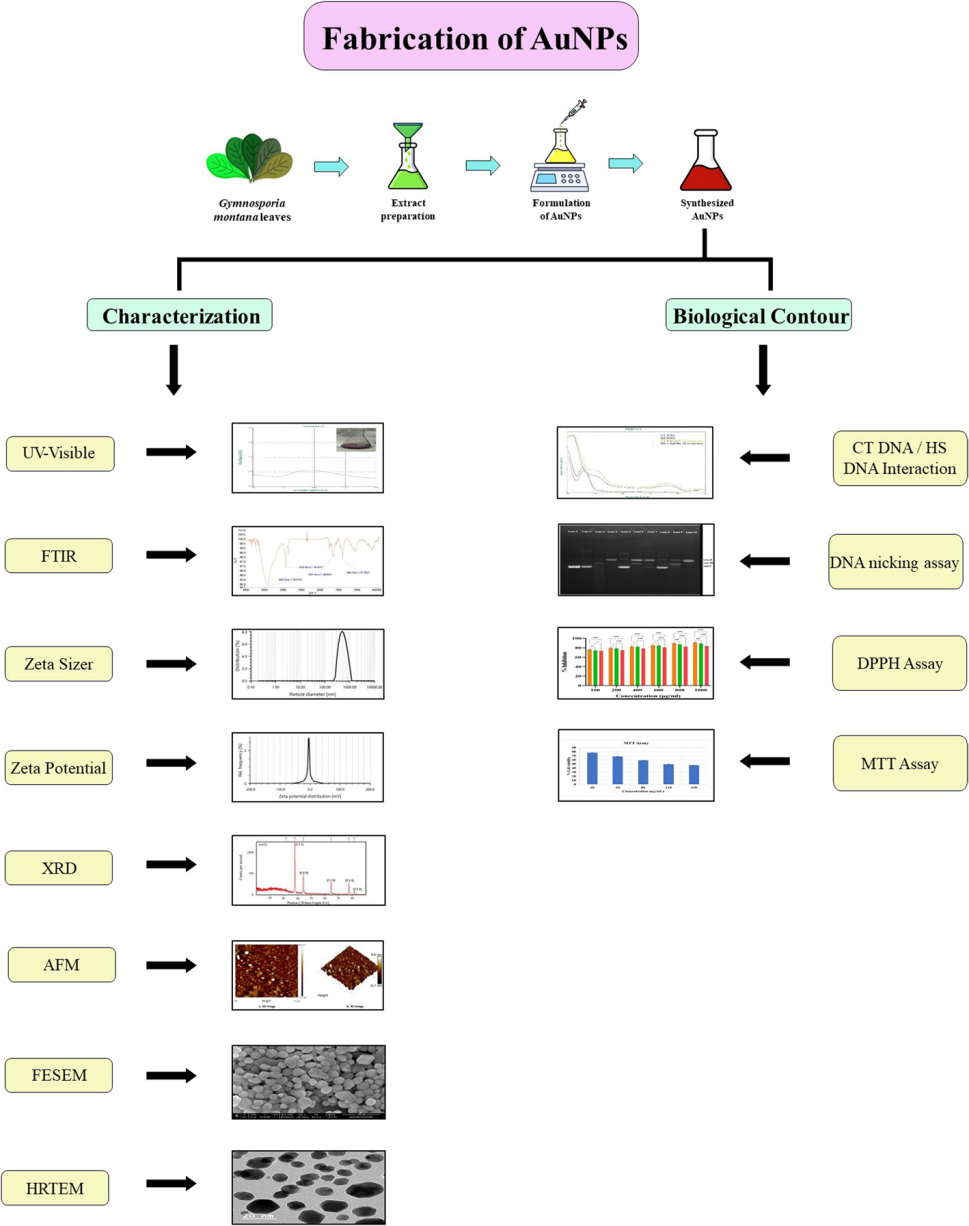

## Background

Nanotechnology and nanoscience are involved in the blending, forming, and characterization of materials and devices whose nanoscopic functional organization, in at least one dimension, is on the nanometer scale or one billionth of a meter [1, 2]. Their distinct size, shape, and larger surface area-to-volume ratio-dependent properties make these materials essential and predominant in many areas of humankind. Metal nanoparticles, predominantly gold nanoparticles (AuNPs), have drawn researchers’ attention more and more throughout the past century and are now widely used in magnetic separation, targeted drug delivery, deoxyribonucleic acid labeling, preconcentration of target analytes, and vehicles for gene and drug delivery, but more significantly, diagnostic imaging due to its ability to interact with light via surface plasmon resonance (SPR), oxidation resistance, and biocompatibility qualities [3–6].

With the two major strategies, top-down and bottom-up nanotechnology conception can be facilitated. The top-down approach involves processing larger things (100–1000 nm) as building blocks to create nanoscale structures (10–100 nm) [7], as opposed to the bottom-up approach, which uses nanotechnology to make larger nanoparticles (NPs) (10–100 nm) from smaller (0.1–10 nm) building blocks like atoms and molecules [8, 9].

Physical, chemical, and biological techniques can be used to produce these NPs from various routes. NPs that have been chemically created are hazardous to living things, which goes against their naturally economical and environmentally favorable nature [10]. Therefore, mounting a greener synthetic approach to produce NPs utilizing biological resources provides a distinctive propitious feature in investigation due to its effectiveness in synthesizing safe compounds [11]. Green substrates such as fungus [12], enzymes [13], microbes [14], algae [15], plants [16], etc. were reportedly successful in the production of AuNPs. However, plant-based synthesis is often risk-free, quick, and operates under ideal room conditions without the need for extreme physical requirements [17]. The selection of appropriate types of protecting agent and concentration, together with synthesis conditions such as pH, temperature, stirring, etc., and the application of optimum reduction and agitation methods may influence the form and size of the AuNPs [18].

*Gymnosporia montana* L. (*G. montana)* commonly known as Viklo occurs throughout the arid, dry areas of India and is traditionally claimed to be useful in various ailments [19]. *G. montana* is a tall shrub with young branches often spiny, bearing leaves, and flowers. Leaves are gray, leathery, obovate, blunt, and measuring 0.5–4.5 × 0.4–2 cm. In addition to evaluating its leaf extract’s potential for anti-inflammatory and hepatoprotective action, researchers have also looked at its historical claims of use in the treatment of inflammation, gastrointestinal disorders, ulcers, and jaundice [19]. The principle phytoconstituents of *G. montana* leaves include phenols, alkaloids, flavonoids, tannins, and saponin [20]. Thus, due to the presence of these potential phytoconstituents, the *G. montana* leaf extract exhibits anti-oxidant, anti-inflammatory, and anti-cancer properties [21]. Thus further, it can be introduced into the gold mixture throughout the NPs fabrication process for the formation of AuNPs.

Biological genetic factory, i.e., deoxyribonucleic acid (DNA), has been a focus of many multidisciplinary research efforts involving nanotechnology. AuNPs are more selective for single-stranded DNA (ss-DNA) binding. Many studies have concluded that electrostatic forces between the anionic DNA strands and the negatively charged surfaces of AuNPs are less favorable for double-stranded DNA (ds-DNA) binding [22, 23]. Henceforth, spectroscopic interactions between the AuNPs produced by *G. montana* with calf-thymus (CT-DNA or ds-DNA) and Herring fish sperm (HS-DNA or ss-DNA) DNA can be performed. DNA nicking assay based on Fenton’s reaction can be applied to analyze the interaction of AuNPs with pBR322 DNA to evaluate the antioxidant strength of the AuNPs. The technique mimics the in vivo biological situation, by producing hydroxyl free radicals from endogenous entities like intracellular iron [24, 25]. Moreover, in vitro chemical antioxidant assay, 2,2-diphenyl-1-picrylhydrazyl (DPPH), can also be carried out for further investigation. Thus, this parameter will assist us in researching and developing nanoparticle-based DNA-binding technology due to its special size and optical characteristics [26].

Although AuNPs have shown potential in a variety of biological applications, a determination of their biosafety is necessary before they can be used more widely [27]. As there is an increasing need to assess how these materials may affect human health and to learn more about their toxicity and biocompatibility, cytotoxicity in vitro assays like MTT (3-(4,5-dimethylthiazol-2-yl)-2,5-diphenyl-2H-tetrazolium bromide) has emerged as the gold standard for determining cell viability and proliferation [28].

By following the same paradigm, we have strained the experiment of synthesizing AuNPs through a biogenic process. To the best of our knowledge, no research using *G. montana* in the manufacture of AuNPs has been published. Here, we used hydrogen tetrachloroaurate (III) trihydrate (HAuCl_4_ · 3H_2_O) and aqueous leaf extract of *G. montana* to demonstrate the biosynthesis, characterization, and biological application of AuNPs*.*

## Methods

### Collection and identification of the plant

**Fig. 1:**
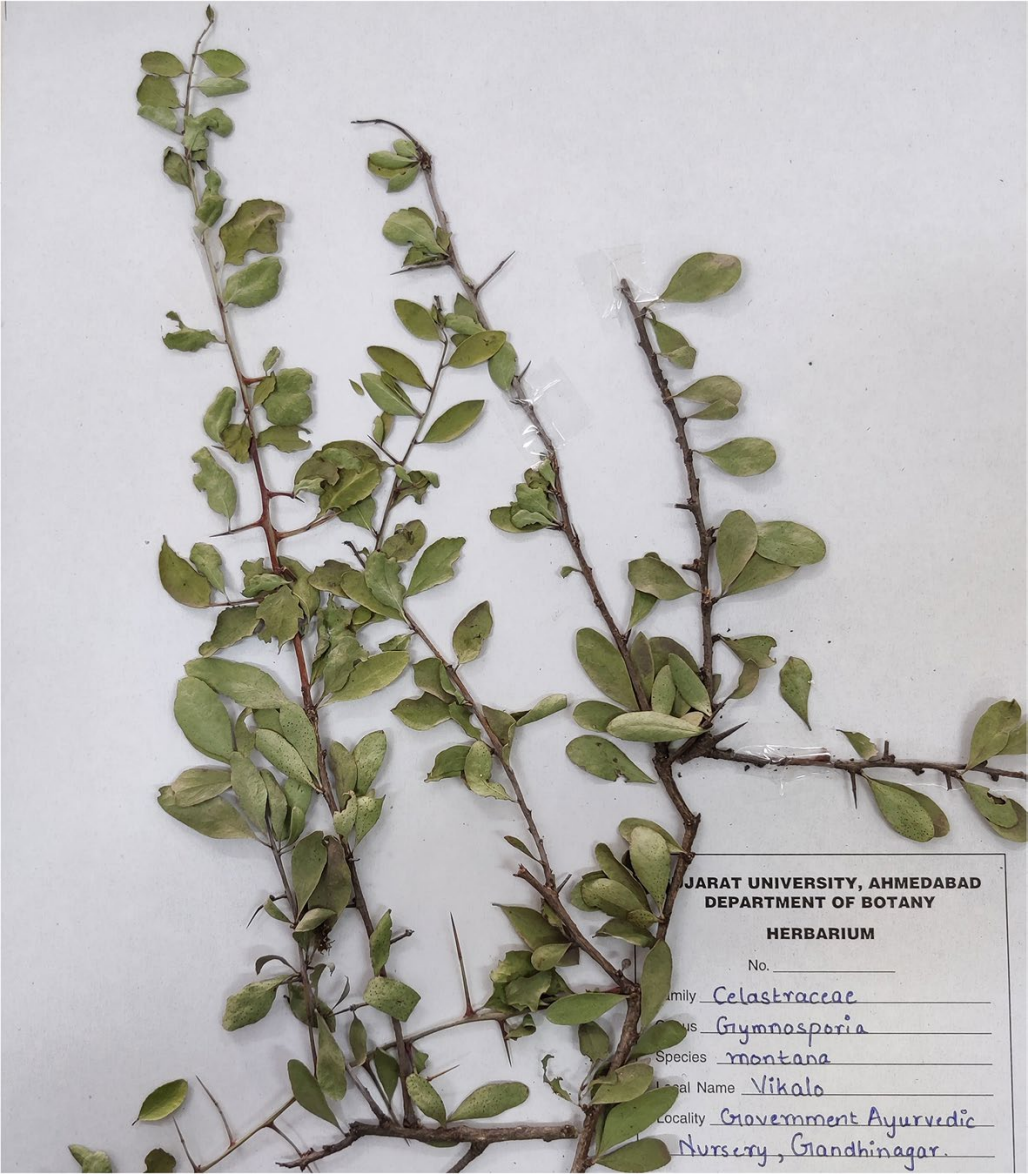
*Gymnosporia montana* L. (Viklo) plant

Indian medicinal plant *G. montana* (Fig. 1) leaf was selected from Gujarat, India, based on its medicinal properties and ease of availability. Fresh (disease-free) and fully expanded healthy leaves of *G. montana* were collected from Government Ayurvedic Nursery, Gandhinagar. It belongs to the *Celastraceae* family. The plant was taxonomically identified and authenticated by the Department of Botany, Bioinformatics, and Climate Change and Impact Management, Gujarat University.

Authenticator: Dr. Hitesh Solanki.

Authentication No.: GU/BOT//C/G 05.

The plant was also identified by the Botanical Survey of India, eFlora Home (Government of India, Ministry of Environment, Forest & Climate Change).

### Preparation of G. montana aqueous leaf extract

After collection, leaves were washed thoroughly with tap water to remove dirt and washed again with distilled water (DW) before being shed-dried for around 8–10 days. Once dried, all the leaves were ground into a fine powder using an electric blender (LG, India) and stored in a well-labeled airtight container at room temperature for further use. The 10% aqueous leaf extract was prepared by weighing 10 gm of the fine powder with 100 ml DW and boiled at 60 °C for 60 min on a magnetic stirrer (REMI, India). The crude extract was filtered with Whatman filter paper no. 1 extract was then stored in the refrigerator (LG, India) at 2–4 °C.

### Biosynthesis of AuNPs

**Fig. 2:**
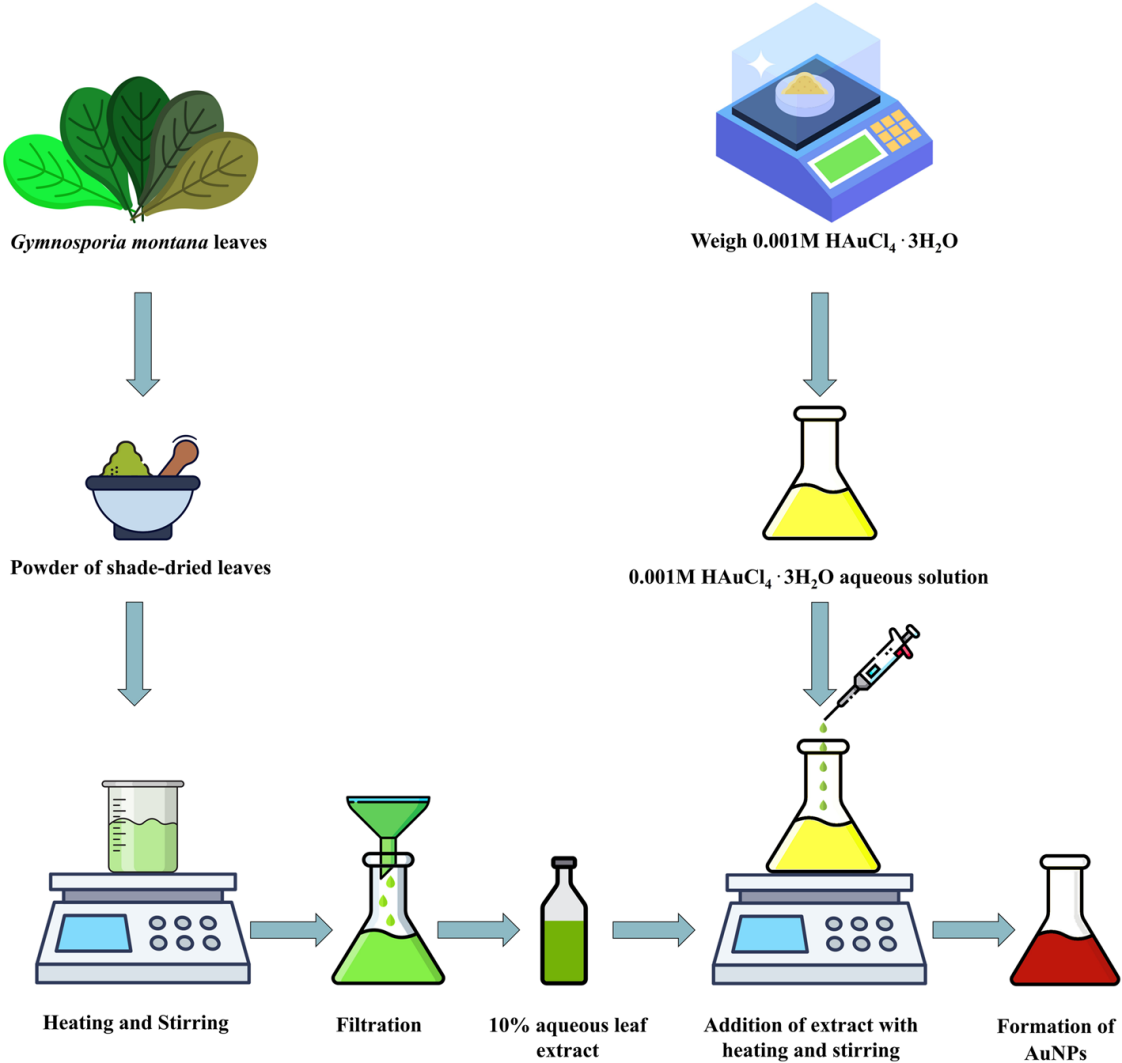
Schematic representation of biosynthesis of AuNPs

In a conical flask, 0.001 M HAuCl_4_ 3H_2_O solution in DW was heated for 30 min at 70 °C. Drop-by-drop 10% aqueous leaf extract of *G. montana* was added under stable and continuous stirring until the solution became yellow to reddish-purple color, confirming the synthesis of AuNPs (Fig. 2). Here, the leaf extract reduces the gold ions and accelerates the formation of AuNPs. The preparation process followed the mechanism of the bottom-up approach. The reduction of HAuCl_4_ · 3H_2_O to AuNPs was monitored periodically with UV–visible spectrophotometer double beam LI-2800 (Lasany, India). In addition, the formed AuNPs were collected by centrifugation at 2000 rpm for 20 min and dried in a hot air oven at 70 °C and stored in a plastic sample vial with appropriate labeling for future use.

### Characterization of AuNPs

#### Ultraviolet–visible spectroscopy analysis

By scanning the absorption maxima with quartz cuvettes with a 1-cm path length and DW as a reference and recording them at wavelength 200–800 nm periodically in a UV–visible spectrophotometer, the reduction of AuNPs was verified. AuNPs plasmon resonance is typically ascribed to an absorption band between 520–600 nm [29].

#### Fourier transform infrared (FTIR) spectroscopy analysis

The dried AuNPs were ground with the KBr pellets with the help of a KBr press, Model M-15, Technosearch instruments [30] and analyzed by FTIR (PerkinElmer Spectrum Version 10.4, USA) in the wavenumber frequency ranging from 4000 to 400 cm^−1^. The FTIR analysis was used for the identification of functional groups present on it which are responsible for bio-reduction of HAuCl_4_ · 3H_2_O. All the dimensions were recorded in transmittance mode.

#### Zeta sizer and zeta potential

Dynamic light scattering (DLS) is an analytical technique for measuring submicron-sized particles’ size distribution and zeta potential. The size and potential aliquots of AuNPs were analyzed using zeta sizer Nano ZS90 (Malvern Instruments, UK) in a disposable cell at 25 °C using Zeta sizer 7.13 software after 5 min of sonication to avoid aggregation of particles.

#### X-ray diffraction (XRD) analysis

XRD (PANalytical X’pert Powder, UK) at 40 mA, 40 kV with CuKα (copper K-alpha) radians at 2*θ* angle (peak position) was used to determine the size, crystalline structure, and chemical composition of the purified AuNPs. The Scherrer equation was used to calculate the mean sizes of the AuNPs using:

$$D=\frac{K\lambda }{\beta\,\mathrm{cos}\theta }$$

where *D* denotes the diameter of the NP (nm), *λ* is the wavelength of the X-ray source (0.15406), *K* is the Scherrer constant (0.9) [31], and *β* is the angular width at FWHM (full width at half maximum) of the diffraction peak Bragg’s diffraction angle *θ* [32].

#### Atomic force microscope (AFM) analysis

AFM typically utilizes a cantilever with a sharp probe to scan a specimen surface [33–35] which helps in providing three-dimensional (3-D) surface profiles. AuNP surface morphology was reviewed using Bruker Nanoscope 8 multimode AFM (USA). Here, first, the AuNPs were adhered to magnetic pucks, using sticky tabs. Next, a liquid solution was dispensed on AuNPs for 2–3 min to enable adsorption and wick away excess solvent. Once adhered, AuNPs were coated using glass or silicon wafers and imaged using open nanoscale analysis.

#### Field emission scanning electron microscope (FESEM) analysis

FESEM is a cutting-edge device that offers extensive details on the composition, shape, and structure of the materials being researched. It produces a range of signals at the surface of solid materials using a concentrated beam of high-energy electrons [36]. Here, gold palladium was used to create a thin layer of AuNPs to make it current-conductive. To prevent surface tension from damaging the tiny structures, it was dried below the critical point. Then, using a Nova NanoFESEM (FEI, USA), it was mounted in the sample holder of a SEM vacuum and was examined.

#### High-resolution transmission electron microscope (HR-TEM) analysis

HR-TEM was used for studying the size and morphology of AuNPs. A drop of the sonicated aqueous suspension of AuNP has dropped on a carbon-coated copper HR-TEM grid. The AuNP-coated copper grid was placed inside the specimen chamber after drying it below the IR (infrared) lamp at room temperature. Shortly after drying, images were taken at different magnifications on JEOL JEM 2100 PLUS high resolution 0.14 nm (Japan), operated at 200 kV HR-TEM. The captured images were analyzed with ImageJ software.

#### Spectroscopic studies of DNA interaction with AuNPs

Here, a homogenous solution was created by dissolving CT-DNA (50 µg/ml) and HS-DNA (33 µg/ml) in injection water and storing the mixture at 5 °C for 24 h. Using a nanophotometer (Implen, USA) and the molar coefficient Ɛ260 = 6600 cm^−1^ M^−1^, the final concentration of DNA solutions was quantified and evaluated qualitatively. The UV–visible spectrophotometer (200–800 nm) was used to record the spectrum of CT-DNA and HS-DNA after the addition of manufactured AuNPs [37].

#### In vitro DNA nicking assay

DNA damage protective activity of AuNPs was investigated using an in vitro DNA nicking assay based on the Fenton reaction. It is an effective approach for assessing biological products’ antioxidant capacity, enabling a quick screening of any genomic damage. As a target DNA in this instance, pBR322 (double-stranded circular) was utilized; 30 mM H_2_O_2_ (hydrogen peroxide), 50 µM ascorbic acid, and 80 µM FeCl_3_ (ferric chloride) were mixed to create the Fenton reagent. DNA was added to 10 µl of AuNP suspension and incubated at 37 °C for 60, 90, and 120 min. Then, 10 µl of Fenton’s reagent was added and incubated again at 37 °C for 60, 90, and 120 min. After incubation, loading dye was added to the reaction solution, and the mixture was run on a 50 V 1% agarose gel in TAE (tris–acetic acid-EDTA) buffer (GeNei, India). After electrophoresis, the gel was examined and captured on camera utilizing the BioDoc-it™ imaging system, UVP.

#### DPPH radical scavenging activity

AuNPs were allowed to react with a stable radical, 2,2-diphenyl-1-picrylhydrazyl (DPPH) in a methanol solution to evaluate its antioxidant activity [38]. In a nutshell, the DPPH solution of 6 × 10^−5^ M was prepared in methanol, and 300 μl of this solution was added to AuNPs and plant extract of *G. montana* (100–1000 μg/ml). After shaking briskly, it was allowed to stand at room temperature for 30 min. As a control, a 1:1 solution of methanol/DPPH was utilized. Ascorbic acid was used as a positive control. All the tubes were in triplicates. A reading was inspected using an EPOCH microplate spectrophotometer by BioTek Gen5™ software at 520 nm using methanol as a blank. Further, the scavenging activity was determined by calculating %inhibition [(A0 – A1)/ A1] × 100, where A0 represents the absorbance of the control and A1 represents the absorbance of the sample. The results were expressed as the mean ± SD. IC_50_ values were obtained using different concentrations of AuNPs.

#### Cytotoxicity assessment using MTT assay

Cytotoxicity evaluation of AuNPs was performed using MTT assay as described by Mosmann [28]. MCF-7 cell line was grown in 10% complete media (10% FBS + 90% DMEM) and was incubated in humidified conditions at 37 °C (carbon dioxide incubator by Eppendorf CellXpert) until the confluency was achieved. Approximately 1 × 10^4^ cells were seeded in each well of 96 well plate. After seeding, the treatment of AuNPs respectively was given in concentrations of 20, 50, 80, 110, and 140 μg/ml. MTT with concentration (5 mg/ml in PBS) was used at a volume of 50 μl was added after 24 h of treatment. After 3–4 h of incubation with MTT reagent, the wells were observed for the presence of formazan crystals which were later dissolved in DMSO at a volume of 100 μl. All the tubes were in triplicates. Utilizing a microplate reader, the absorbance was measured at 570 nm. The %viability of all the samples was calculated using the following formula: %viability = (sample O.D/control O.D) × 100. The results were expressed as the mean ± SD. IC_50_ values were obtained using different concentrations of AuNPs.

#### Statistical analysis

The statistical analysis was conducted using GraphPad Prism, Version 8, utilizing the two-way ANOVA with repetition technique. The *p*-values obtained from the analysis were interpreted as follows: a *p*-value less than 0.05 (*) indicated a probability of less than 5% that the observed effect occurred by random chance, a *p*-value less than 0.01 (**) indicated a probability of less than 1%, a *p*-value less than 0.001 (***) indicated a probability of less than 0.1%, and a *p*-value less than 0.0001 (****) indicated a probability of less than 0.01%. Conversely, when the observed results or difference were not statistically significant, the term “ns” (non-significant) was used to indicate that the observed effect was likely due to random chance.

## Results

### Characterization of AuNPs

#### UV–visible spectroscopy analysis

**Fig. 3:**
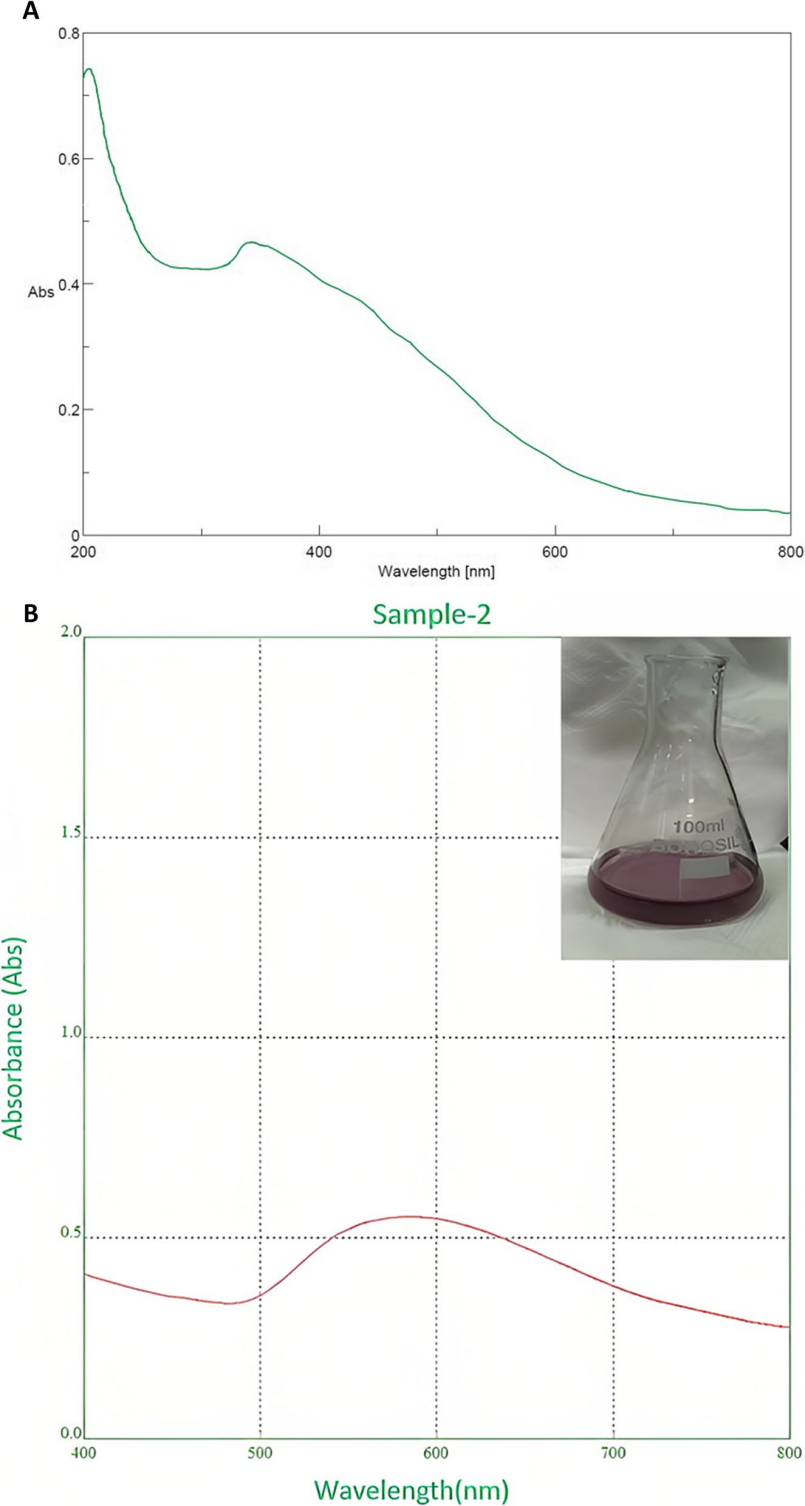
**a** UV–visible spectrum of *Gymnosporia montana.* **b** UV–visible spectrum of AuNPs

During monitoring of the progress of the reaction, it was observed that the initial color of HAuCl_4_ · 3H_2_O changed from yellow to reddish pink with the wavelength maximum (*λ*_max_) absorbance near 595 nm (Fig. 3b), which indicated the generation of AuNPs, and the intensity of the color was increased with incubation time. Also, Fig. 3a shows the absorbance peak of around 380 nm for bare leaf extract of *G. montana.*

#### FTIR analysis

**Fig. 4:**
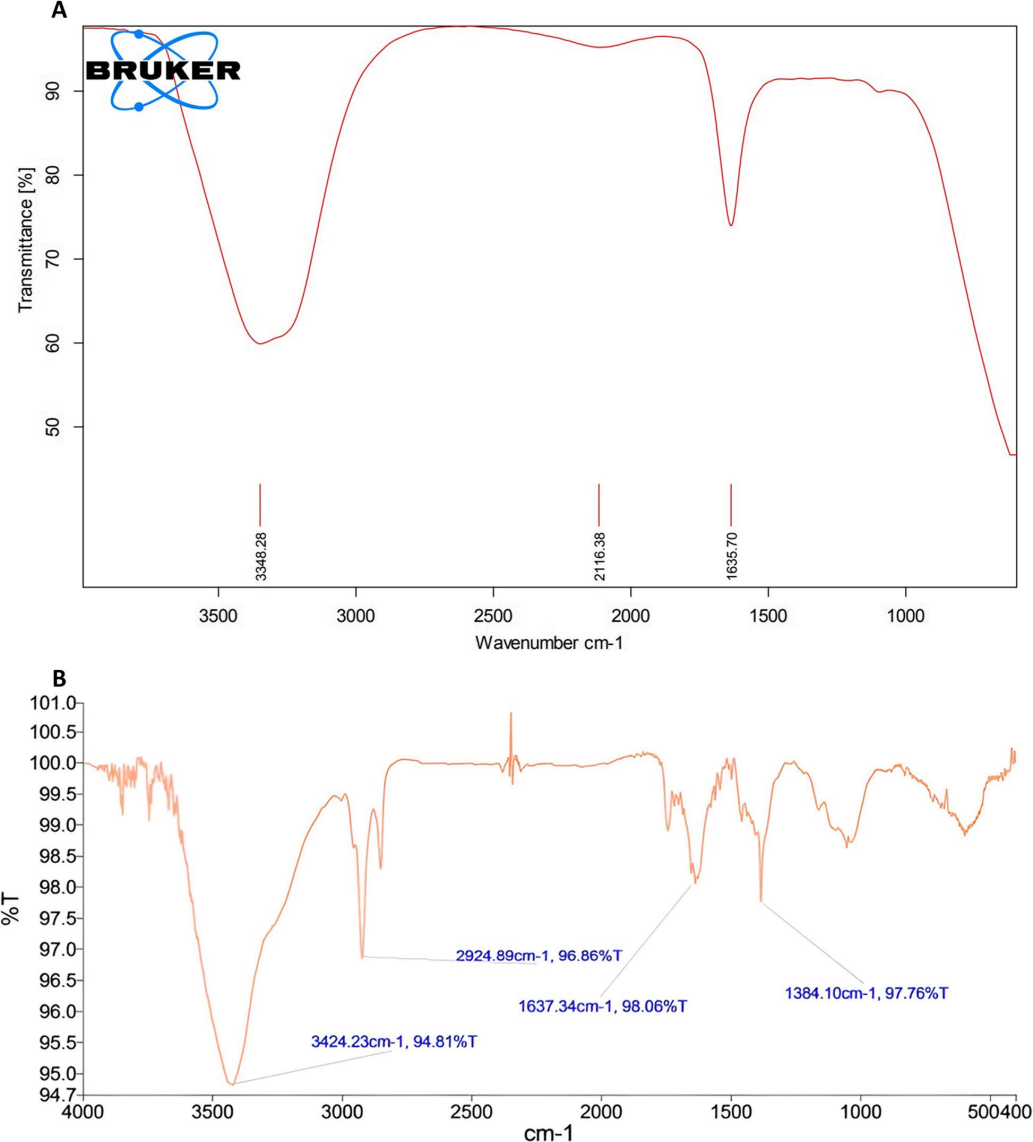
**a** FTIR spectrum of *Gymnosporia montana.* **b** FTIR spectrum of AuNPs

FTIR serves as the molecular fingerprinting for the identification of organic materials. FTIR analysis was conducted to characterize the functional group of *G. montana* leaf extract which may be responsible for the reduction, capping, and stability of AuNPs. FTIR spectra (Fig. 4a) showed peaks of *G. montana* leaf extract at 3348.28 cm^−1^ representing O–H stretching, 2116.38 cm^−1^ representing C–H stretching of alkanes, and 1635.70 cm^−1^ representing -C = C- stretching of alkenes. Figure 4b showed peaks at 3424.23 cm^−1^ representing O–H stretching of phenols and alcohols; 2924.89 cm^−1^ indicates the presence of C–H stretching vibration of alkenes; 1637.34 cm^−1^ indicates -C = C- stretching of alkenes; 1384.10 cm^−1^ indicates N–O stretching of nitro compounds, suggesting the presence of flavanones or terpenoids adsorbed on the surface of AuNPs.

#### Zeta sizer and zeta potential analysis

**Fig. 5:**
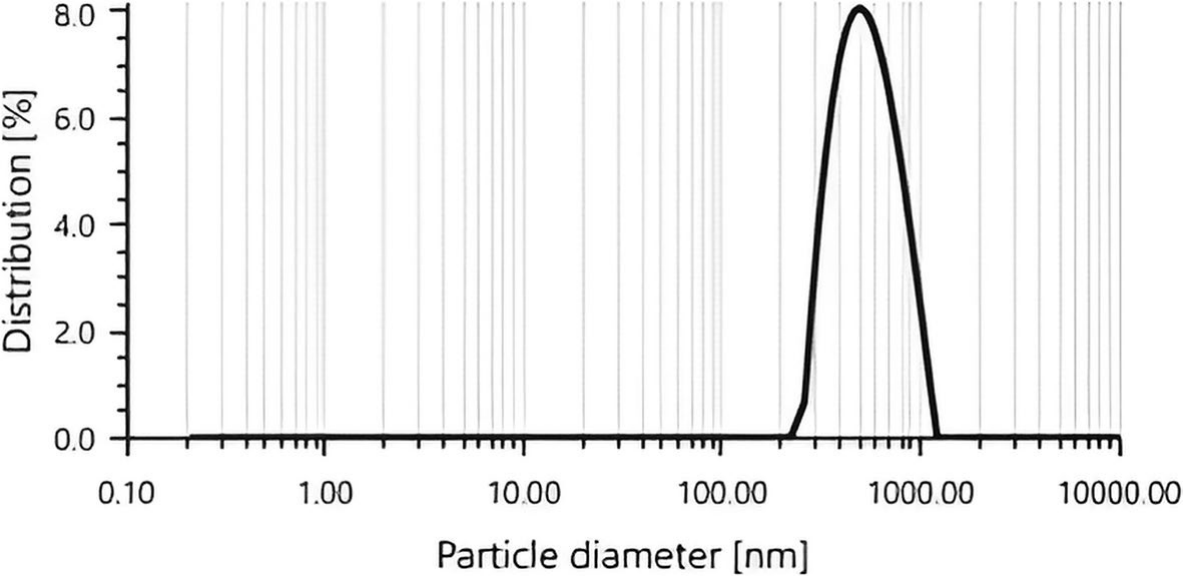
Zeta sizer of AuNPs

**Fig. 6:**
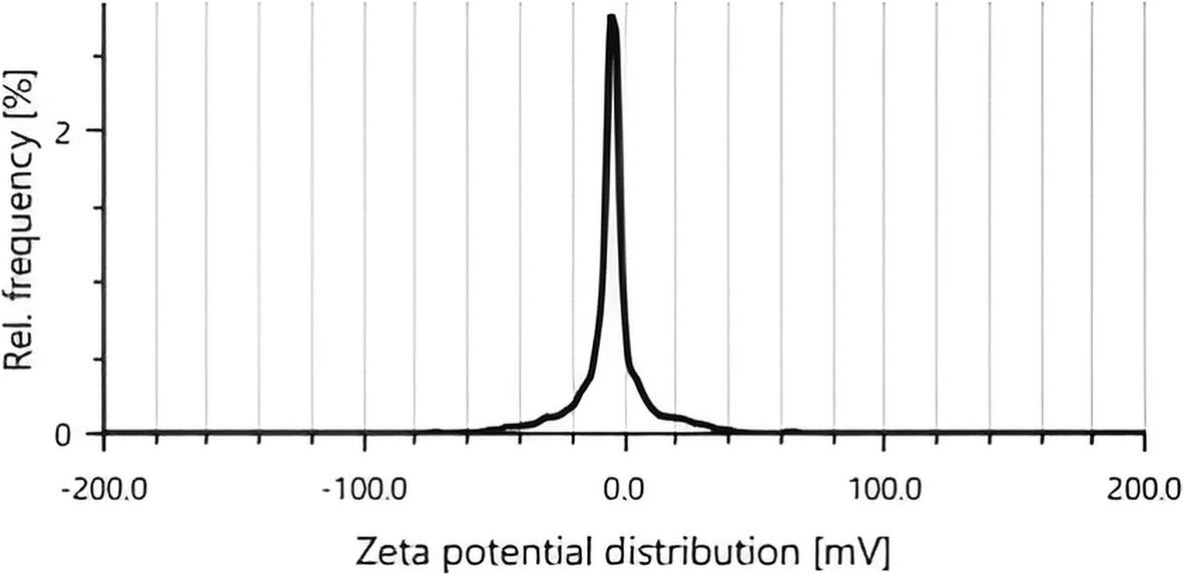
Zeta potential of AuNPs

The size and size distribution of the *G. montana*-produced AuNPs is depicted in Fig. 5 since these two characteristics are crucial to understanding both their physical characteristics and potential uses. With a polydispersity index (PdI) of 11.3%, the particle size distribution measured by the zeta sizer was 559.6 d. nm AuNPs’ zeta potential, which was measured at − 4.5 mV, demonstrated stability (Fig. 6).

#### XRD analysis

**Fig. 7:**
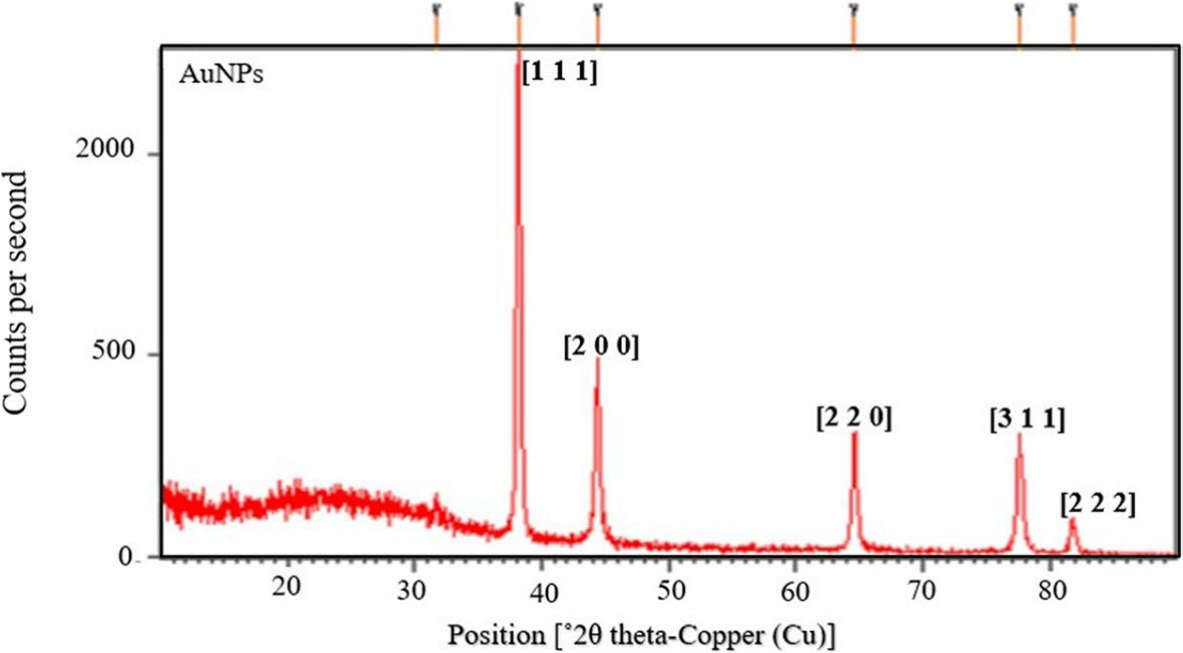
XRD analysis of AuNPs

XRD is used for characterizing NP’s crystalline structure because of its ability to interact with electrons of the inner shell of an atom. Here, the XRD spectrum of *G. montana* synthesized AuNPs (Fig. 7) spanned from 10° to 80°. The AuNPs were in nanostructures, as evidenced by the Braggs’ diffraction peaks at 2*θ* values of 38.1459°, 44.3725°, 64.5529°, 77.5352°, and 81.7492° corresponding to (111), (200), (220), (311), and (222) identical planes with those reported for the standard gold metal Au^0^ (joint committee on powder diffraction standards-JCPDS no. 04–0784, USA) [39]. The respective diffraction peaks relate to the facets of the face-centered cubic (FCC) crystal lattice. The width of the planes was used for the determination of crystallite size. Using the Debye–Scherrer equation, the mean size of AuNPs was calculated, where *d* = particle size, *λ* = wavelength of the radiation, i.e., 0.154 nm, *θ* = angle of Bragg plane, i.e., 38.1459°, *β* = angular width at FWHM, i.e., 0.2657, and *K* = the Scherrer constant. The average crystalline size was found to be ranging from 10 to 50 nm.

#### AFM analysis

**Fig. 8:**
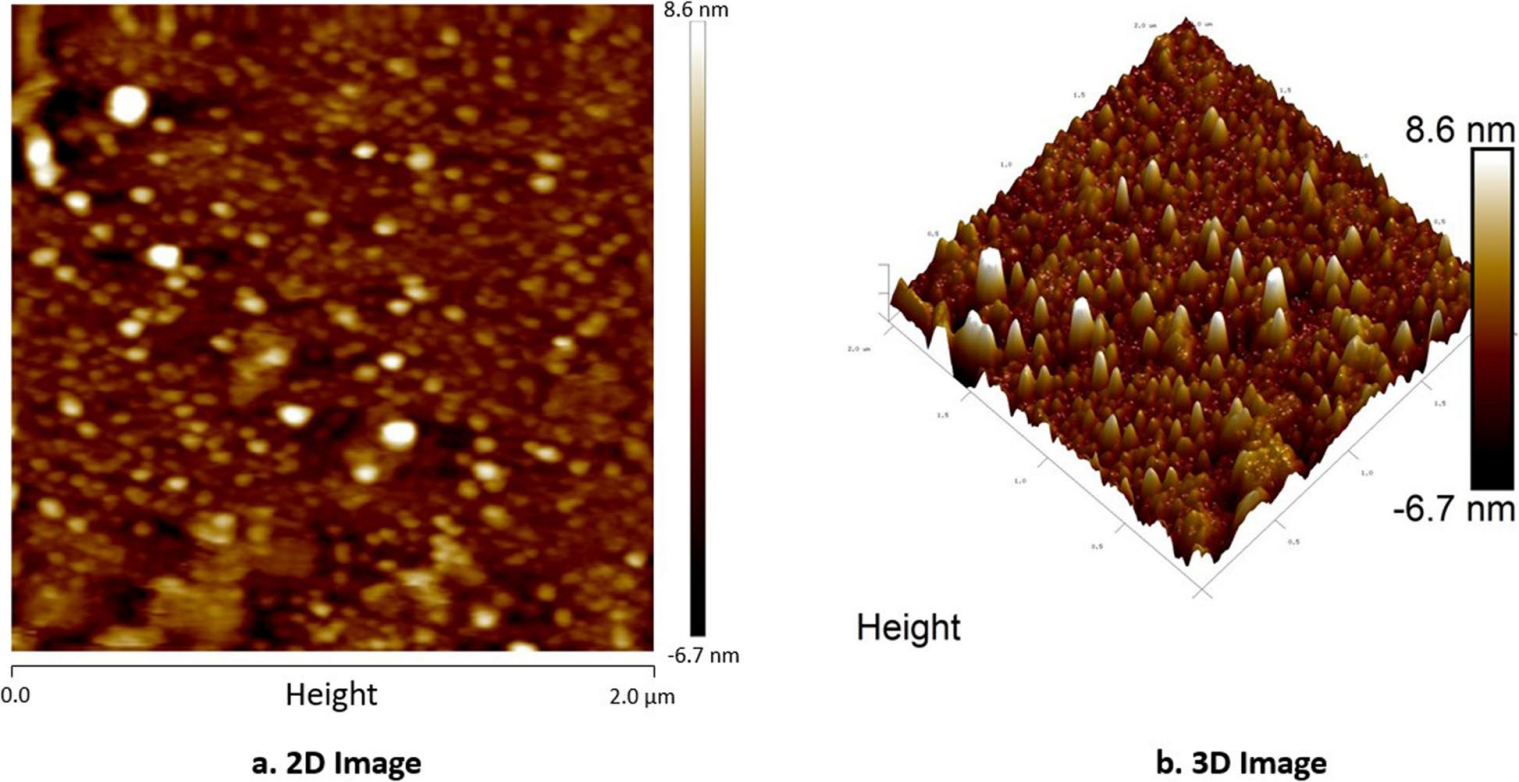
AFM analysis of AuNPs. **a** 2D image. **b** 3D image

With sub-nanometer resolution, AFM enables the 3D characterization of NPs. Here, the AuNPs with irregular-shaped morphology and 6.48 nm particle size are shown in the 2D and 3D images in Fig. 8a and b.

#### FESEM analysis

**Fig. 9:**
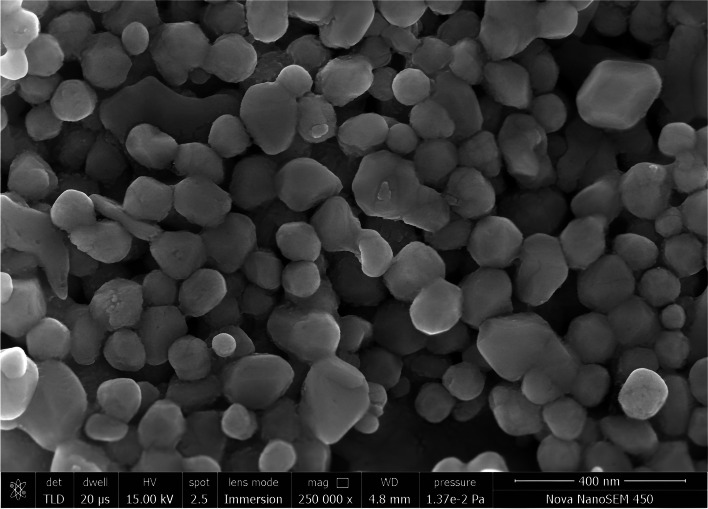
FESEM analysis of AuNPs

**Fig. 10:**
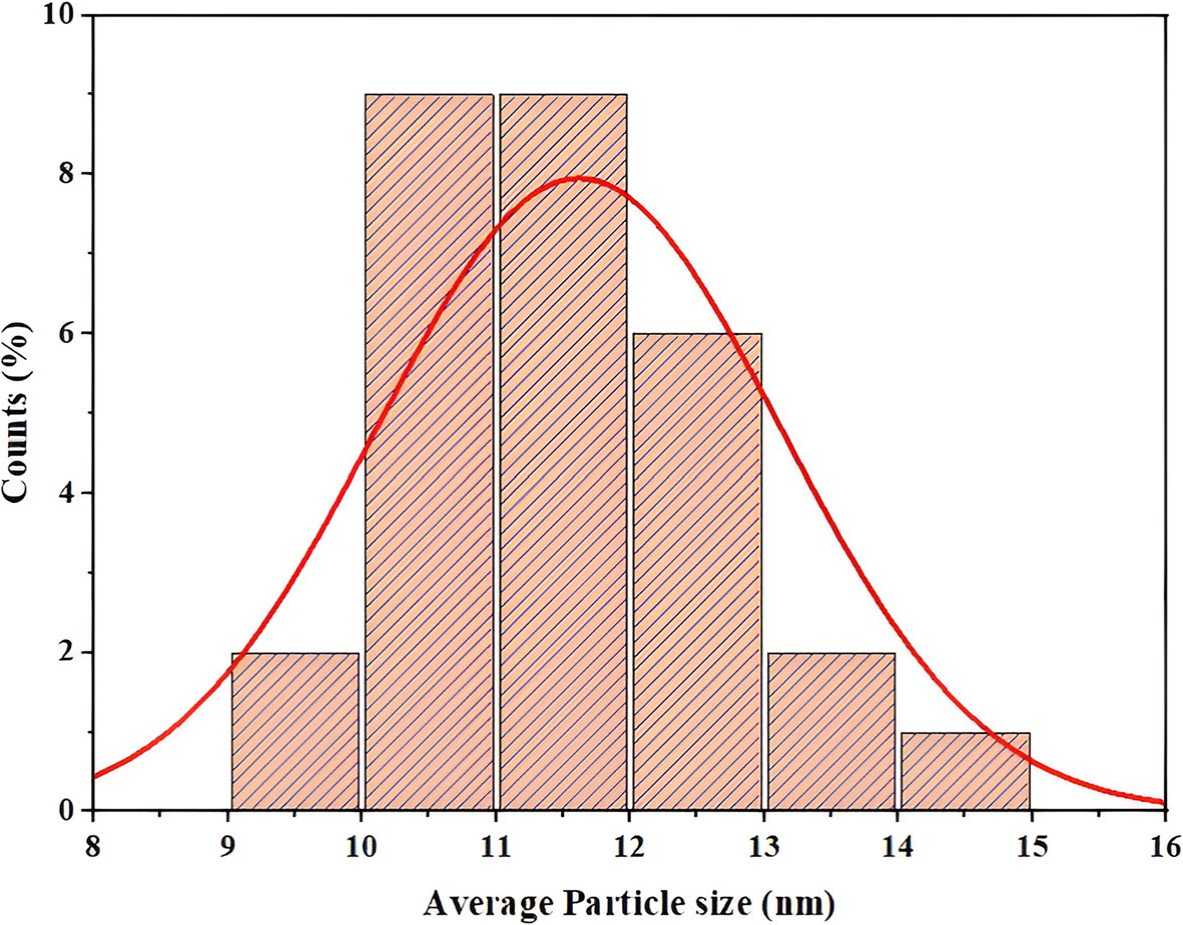
Histogram analysis of AuNPs (FESEM)

FESEM study of the AuNPs produced by *G. montana* is shown in Fig. 9. Here, the AuNPs primarily showed a spherical shape, with diameters between 10 and 20 nm. The AuNPs’ diameters were calculated using ImageJ 1.53t software. Using the OriginPro software version 2023, the histogram (Fig. 10) was plotted for the same data. The average particle size was estimated with the aid of a histogram, and the size that was discovered was 11.61 nm.

#### HR-TEM analysis

**Fig. 11:**
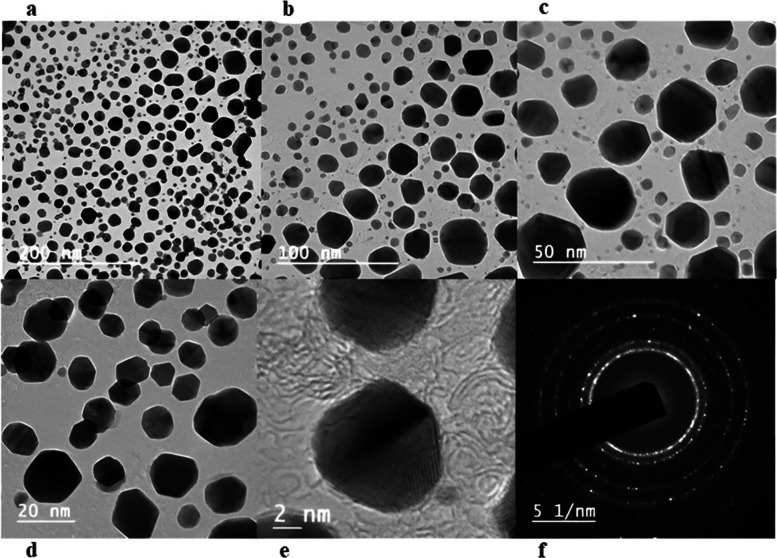
HR-TEM analysis at different resolution. **a–d** HR-TEM analysis at 200, 100, 50, and 20 nm resolution. **e** UHR-TEM at 2 nm resolution and **f** SAED pattern of AuNPs

HR-TEM images confirmed that AuNPs were polydisperse, consisting predominantly of spherical and subsidiarily hexagonal shapes, ranging from 10 to 50 nm with different resolutions at 200 nm, 100 nm, 50 nm, and 20 nm, respectively (Fig. 11a–d). The ultra-high-resolution TEM (UHR-TEM) revealed (Fig. 11e) clear lattice fringes on the particle surface. According to the selected area electron diffraction (SAED) pattern (Fig. 11f), the *d* spacing rings were observed corresponding to (0.237 nm) of (1 1 1), (0.204 nm) of (2 0 0), (0.145 nm) of (2 2 0), and (0.123 nm) of (3 1 1) planes of (JCPDS card no: 04–0784) the FCC crystalline lattice of AuNPs [39, 40].

#### Spectroscopic studies of DNA interaction with AuNPs

**Fig. 12:**
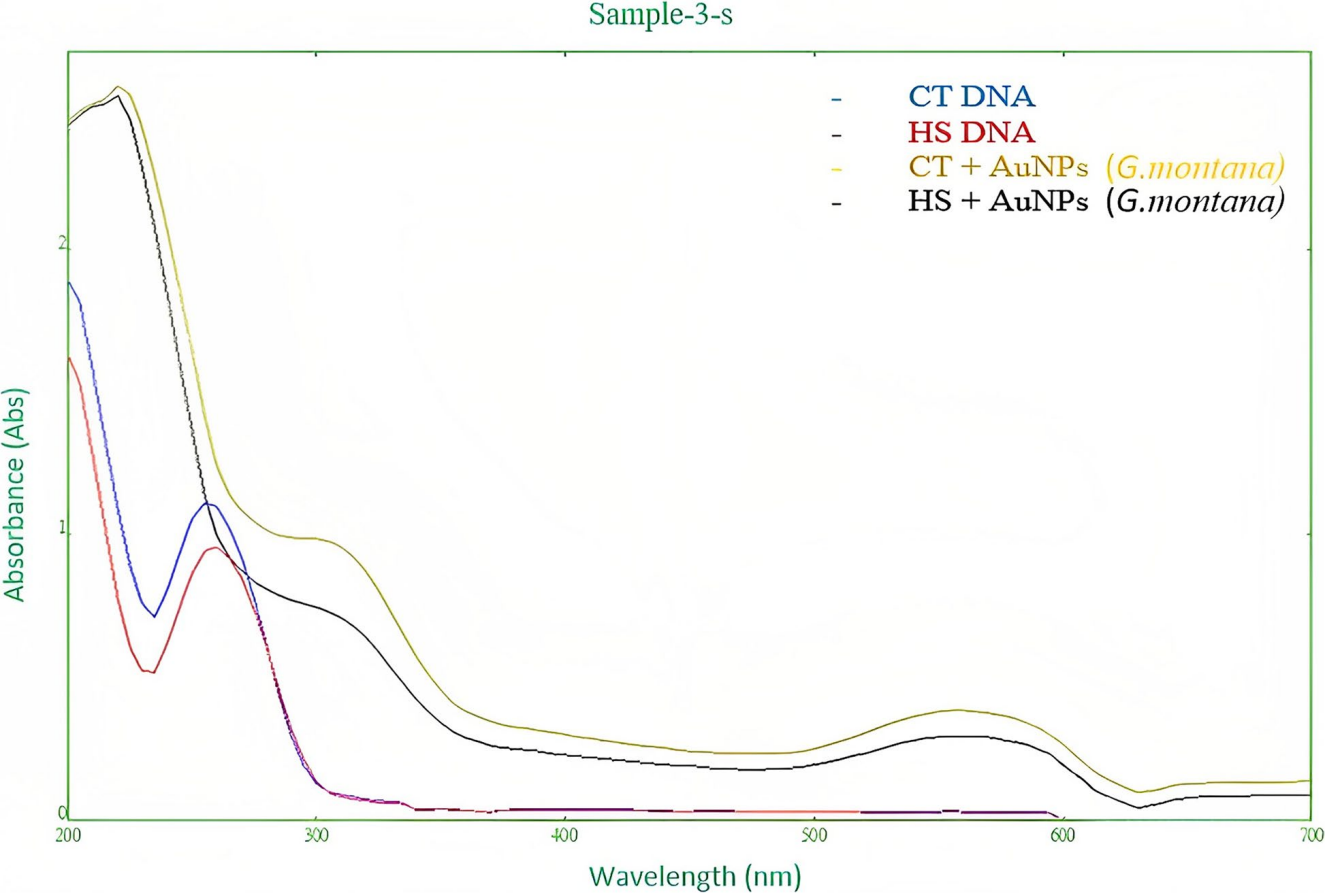
Spectroscopic interaction of DNA with AuNPs

The qualitative and quantitative analysis of CT-DNA and HS-DNA at 260/280 showed 1.667 and 1.72 absorbance ratios, respectively. The absorption spectra interaction of both CT-DNA and HS-DNA with AuNPs showed bathochromic and hypochromic shifts (Fig. 12).

#### In vitro DNA nicking assay

**Fig. 13:**
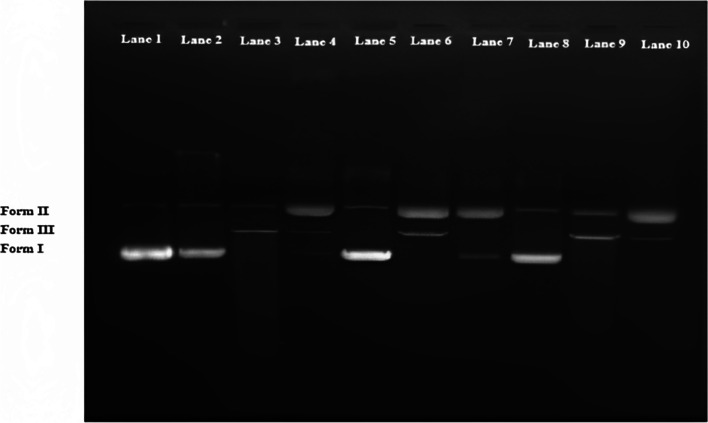
Gel interaction of pBR322 DNA with AuNPs. Lane 1: control pBR322; lane 2: pBR322 + AuNPs (37 °C for 60 min); lane 3: pBR322 + Fenton’s reagent (37 °C for 60 min); lane 4: pBR322 + AuNPs + Fenton’s reagent (37 °C for 60 min); lane 5: pBR322 + AuNPs (37 °C for 90 min); lane 6: pBR322 + Fenton’s reagent (37 °C for 90 min); lane 7: pBR322 + AuNPs + Fenton’s reagent (37 °C for 90 min); lane 8: pBR322 + AuNPs (37 °C for 120 min.); lane 9: pBR322 + Fenton’s reagent (37 °C for 120 min); lane 10: pBR322 + AuNPs + Fenton’s reagent (37 °C for 120 min)

In the DNA nicking assay (Fig. 13) due to **·**OH formation during the reaction, which is highly reactive and strong oxidizing species, the initial supercoiled (form I) configuration of pBR322 DNA changes from supercoiled to open circular (form II) and nicked linear (form III) forms that present altered electrophoretic mobility properties on the gel [41]. Here, pBR322 was exposed to AuNPs and Fenton’s reagent at 37 °C for 60, 90, and 120 min for time-dependent study.

At different incubation durations, AuNPs did not differ from the control (lane 1) in the native form of pBR322 (lane: 2, 5, 8). After 60 min (lane 3), Fenton’s reagent changed the phosphodiester chain in pBR322 DNA from supercoiled (form I) to open circular (form II) and nicked linear (form III), an additional incubation for 90 and 120 min showed more scission with open circular (form II) and nicked linear (form III) (lane: 6, 9). Fenton’s reagent and AuNPs were exposed under identical circumstances, and the results showed that AuNPs were able to preserve DNA from free radicals by protecting form I and form II (lane: 4 and 7) at incubation periods of 60 min and 90 min, whereas DNA was cleaved into form II and form III at an increased incubation period of 120 min (lane: 10).

#### DPPH radical scavenging activity

**Fig. 14:**
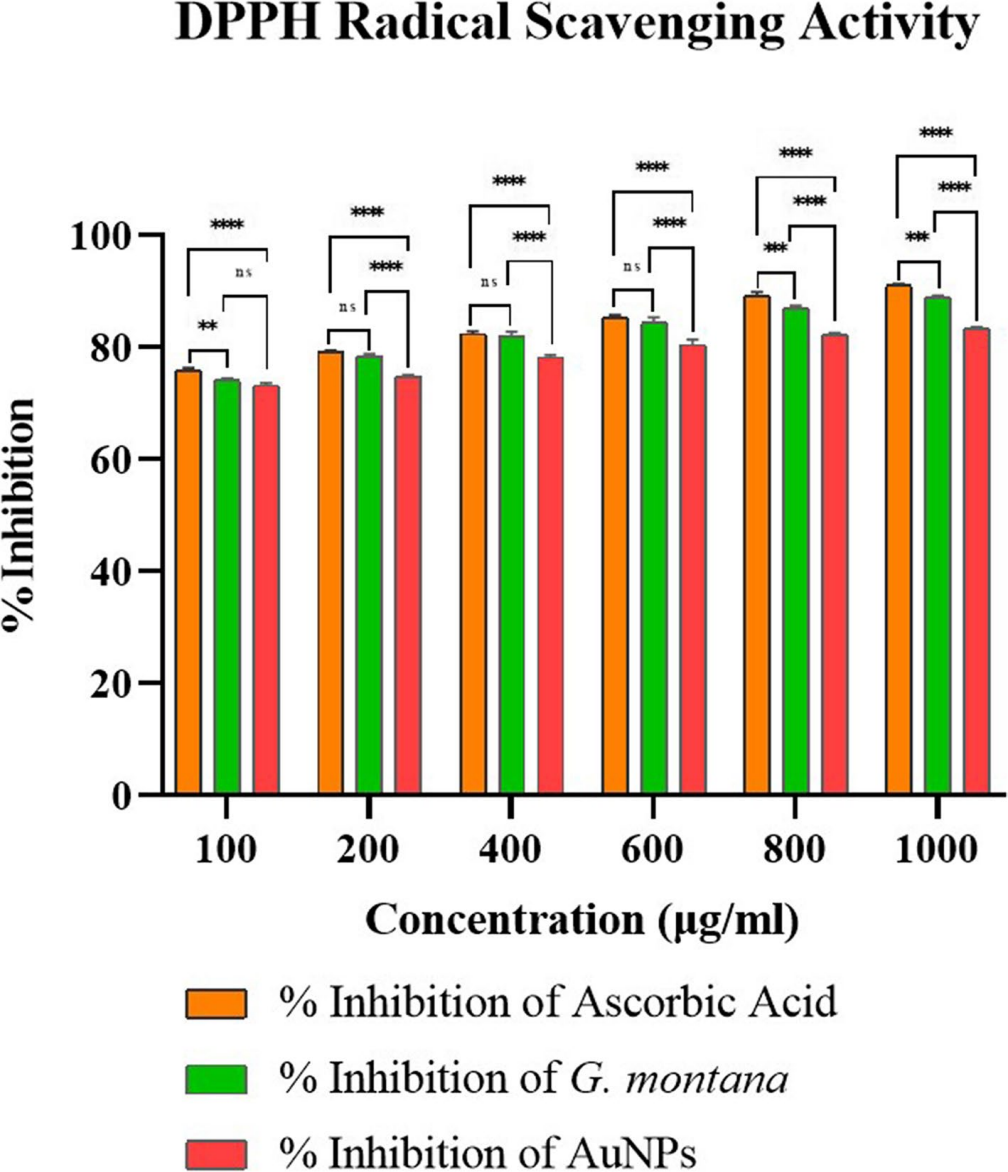
DPPH radical scavenging activity of *G. montana* and AuNPs

The DPPH assay was used to assess the antioxidant activity of *G. montana* and AuNPs. The results demonstrated that both *G. montana* and AuNPs exhibited antioxidant activity, indicating that the antioxidant capacity of *G. montana* was retained in the nanoparticles. The effective percentage inhibition values for ascorbic acid, *G. montana*, and AuNPs were determined as 90.75%, 89.07%, and 83.19%, respectively (Fig. 14). The statistical analysis indicated that *G. montana*, used as a reducing agent for the reduction of AuNPs, showed significant activity comparable to the positive control, ascorbic acid. Furthermore, the statistical significance suggested that both *G. montana* and AuNPs exhibited considerable antioxidant activity at higher concentrations i.e., above 800 μg/ml. The IC_50_ values, which represent the concentration required for 50% inhibition, were determined as follows: ascorbic acid (1503 μg/ml), AuNPs (1959.39 μg/ml), and *G. montana* (1612.35 μg/ml).

#### Cytotoxicity assessment using MTT assay

**Fig. 15:**
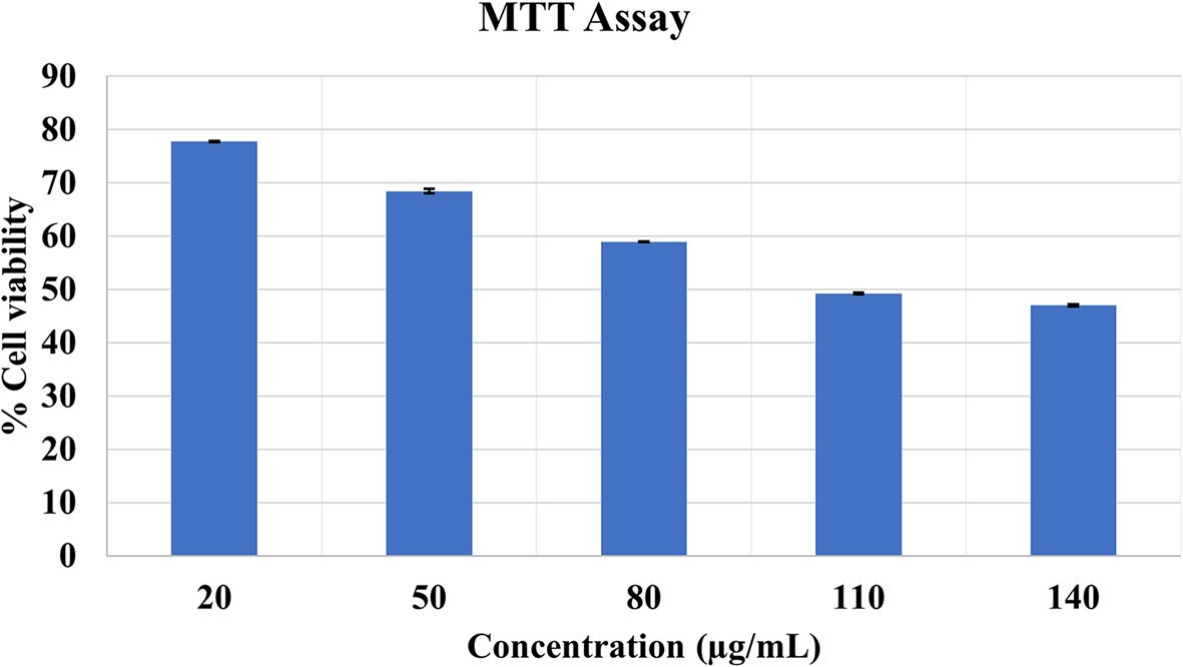
Cytotoxicity assessment of and AuNPs

The cytotoxicity of the synthesized AuNPs was evaluated on MCF-7 cell line. The results showed that *G. montana* synthesized AuNPs caused a dose-dependent reduction in cell viability, as depicted in Fig. 15. The IC_50_ values of AuNPs was determined as 118.13 μg/ml.

## Discussion

The green synthesis process was used to manufacture AuNPs. According to numerous other researchers, the subject of this study discovered that *G. montana* leaf extract could convert Au^+3^ to Au^+0^ as shown by a change in the reaction from yellow to a reddish-pink hue, which indicated the formation of AuNPs [42, 43]. UV–visible spectroscopy was used in this instance as a rapid and easy tool to analyze the bio-redox reaction of HAuCl_4_ × 3H_2_O to AuNPs [44]. According to earlier research, the collective wavering of conduction electrons isolated to metallic NPs causes the localized SPR peak [45]. Thus, UV–visible spectrum’s peak at 595 nm (Fig. 3b) revealed the production of AuNPs from *G. montana*. FTIR spectroscopy was used to classify the biomolecules according to their functional groups, which aided in the stabilization and reduction of the generated AuNPs [46]. The changes in spectral bands resulting from the bio-reduction of HAuCl_4_ 3H_2_O to AuNPs using *G. montana* leaf extract were evident in Fig. 4a and b. The observed shifts were quite significant, with the band at 3348 cm^−1^ shifting to 3424.23 cm^−1^, and a minor shift from 1635.28 to 1637.34 cm^−1^. Various functional groups were identified in the AuNPs, such as alkene vibrations (C–H and -C = C- stretching), nitro compounds (N–O stretching), and phenols/alcohols (O–H stretching). These findings were supported by Folorunso et al., Sett et al., and Xin lee et al. [6, 16, 47, 48], who also reported similar results.

The particle size of the AuNPs was determined to be 559.6 d. nm (Fig. 5) utilizing zeta sizer technique since the correct size and charge are important components of NPs synthesis. Here, large concentrations of HAuCl_4_ × 3H_2_O and/or reducing metabolites caused an increase in NP size, which in turn caused the particles to clump together as evenly specified in Ali et al. 2016 [49]. Zeta potential analysis was used to determine the AuNPs’ long-term stability and surface charge [50]. The generated AuNPs had a zeta potential of − 4.5 mV (Fig. 6). While the positive charge indicates the possibility for aggregation and instability, the negative behavior demonstrates the existence of large electric charges on the particle surfaces, which inhibit impending agglomeration and provide stability [51]. According to Fig. 7, the XRD pattern is in agreement with earlier reports about the produced AuNPs [52] to be crystalline in character, with a considerably stronger peak at 38.1459° indicating the FCC gold structure. Inter planar distance calculated from Bragg’s law was observed at 0.237 nm which agrees with HR-TEM results. Using the Scherrer equation, the mean crystalline size of AuNPs was ranging between 10 and 50 nm [40], and the same was confirmed from the data analyzed from HR-TEM images (Fig. 11a–e), which showed polydispersity, majorly containing spherical and subsidiarily hexagonal shape. The 2D and 3D topography of the AuNPs AFM analysis, with a diameter of 6.48 nm, is shown in Fig. 8a and b, demonstrating that NPs have a larger surface area [52]. FESEM image showed majorly spherical-shaped AuNPs (Fig. 9). According to the results, the particle size ranged between 10 and 20 nm. In this case, the components on the NP’s surface that serve as a capping agent can be the cause of the AuNPs’ aggregation [53].

Spectroscopy is a useful tool for determining how DNA interacts with NPs [54, 55]. Chromism is a spectral characteristic that influences the stability of DNA. Hypochromic and bathochromic complexes indicate that the DNA is bound by electrostatic influence or intercalation, which can stabilize the DNA structure, whereas hyperchromic complexes show that the DNA’s secondary structure has been disrupted [56, 57]. CT-DNA and HS-DNA interaction with *G. montana* synthesized AuNPs resulted in bathochromic and hypochromic shifts (Fig. 12), indicating intercalation property due to a decrease in the energy gap between the highest (HOMO) and the lowest (LUMO) molecular orbitals [58] and non-covalent intercalative binding of the compound to DNA helix, due to strong stacking interaction between the aromatic chromophore of the compound and base pairs of the DNA, respectively [59].

Antioxidant substances can impede the oxidation chain at multiple points in the process, either via chelating catalyst-active metals or by radical-chain breaking. For biologically in vivo antioxidant compounds, an in vitro DNA nicking assay based on the Fenton reagent was carried out. As stated in Leba et. al. [60], DNA nicking assays have been developed because damage to the genome is the primary cause of disorders like cancer and neurological illness [61–63]. With incubation at 37 °C for 60 and 90 min, respectively, the AuNPs produced from *G. montana* in Fenton reagent were able to maintain the pBR322 DNA forms I and II. But when the incubation time lengthened, it split the DNA into forms II and III (Fig. 13). Additionally, because *G. montana*-generated AuNPs can preserve the native form of pBR322, they can be employed as a disease-curing drug. A similar investigation with CPTH (calix [4] pyrrole tetrahydrazide)-AuNPs was carried out by Kongor et al. [64].

Besides, to the in vitro DNA nicking experiment for genomic agents, the DPPH in vitro chemical assay was carried out to assess the overall antioxidant capability of natural products [65–67]. Oxygen interactions with specific molecules can result in the formation of free radicals. As a result, it constantly tries to form stable bonds by obtaining or losing an unpaired electron.

Gold, with its ability to easily gain or lose electrons and modify existing oxidation states, has the potential to strengthen bonds. In accordance with Soshnikova et al., higher doses of gold nanoparticles (AuNPs) have been shown to increase their scavenging activity [68]. When comparing the antioxidant activity of *G. montana* leaf extract to the positive control (ascorbic acid), it was observed that ascorbic acid exhibited a percentage inhibition ranging from 75.97 to 91.06% across concentrations of 100–1000 μg/ml. In contrast, *G. montana* leaf extract showed non-significant results (ns) in relation to ascorbic acid at concentrations ranging from 200 to 600 μg/ml. However, at approximately 800 μg/ml concentration, both ascorbic acid and *G. montana* displayed a significant value of *p* < 0.01 (**) indicating similar scavenging activity against reactive oxygen species. Figure 14 also indicates that concentrations above 800 μg/ml for both *G. montana* and AuNPs exhibited increased antioxidant activity, with significant values of *p* < 0.001 (***). Specifically, the inhibition percentages rose from 73.18 to 83.37% for AuNPs and from 74.16 to 88.96% for *G. montana* leaf extract. To summarize, *G. montana* leaf extract demonstrated comparable antioxidant activity to the positive control (ascorbic acid) at around 800 μg/ml concentration, as indicated by the significant *p*-value. Moreover, concentrations exceeding 800 μg/ml for both *G. montana* and AuNPs showed enhanced antioxidant activity, with statistically significant results. The IC_50_ values obtained from the DPPH radical scavenging activity indicated that the concentration of 50% of the DPPH radicals were scavenged by each substance. Thus, ascorbic acid exhibited the highest antioxidant activity, followed by *G. montana* and AuNPs. These findings suggest the potential of these substances as DPPH scavengers and provide insight into their relative antioxidant capabilities.

The MTT assay results presented in Fig. 15, indicated that both AuNPs coated with *G. montana* leaf extract showed a concentration-dependent decrease in cell viability when exposed to increasing concentrations [69]. These findings highlight the potential cytotoxic effects of higher concentrations of AuNPs on MCF-7 cell line.

## Conclusion

NPs are an innovative form of substance that has surfaced in recent years. The green technique is primarily straightforward to use and is ecologically benign for controlling the size and form of the NPs. AuNP diverse characterization revealed their distinctive optical and SPR properties and surface chemistry, which stated their potentiality in the fields of chemistry, biology, and physics. Additionally, biogenic interactions exhibited stability, intercalative, and antioxidant properties that had outstanding biocompatibility and bioavailability with physiologically active organic molecules. The spectrum of AuNP toxicity suggests appropriate use in a variety of medical specialties in the future. It may also be used for in silico and in vivo experiments for anticancer, gene, or drug delivery agents since the foundation of the *G. montana*-manufactured AuNPs demonstrated significant results.

## Data Availability

All the data generated and investigated in this study are included in this manuscript.

## Abbreviations

°C: Celsius
µg: Microgram
µl: Microliter
µM: Micromolar
µg/ml: Microgram per milliliter
mL^−^^1^: Milli reciprocal liter
M: Molar
mA: Milliampere
V: Volt
kV: Kilovolt
mV: Millivolt
cm: Centimeter
cm^−^^1^: Reciprocal centimeter
cm^−^^1^ M^−^^1^: Reciprocal centimeter reciprocal meter
d.: Diameter
nm: Nanometer
min.: Minutes
hrs: Hours
IC_50_: 50% Inhibitory concentration
SD: Standard deviation
O.D.: Optical density
rpm: Rotation per minute
